# Failing to replicate predicts citation declines in psychology

**DOI:** 10.1073/pnas.2304862120

**Published:** 2023-07-10

**Authors:** Cory J. Clark, Paul Connor, Calvin Isch

**Affiliations:** ^a^The Wharton School, University of Pennsylvania, Philadelphia, PA 19104; ^b^School of Arts and Sciences, University of Pennsylvania, Philadelphia, PA 19104

**Keywords:** psychology, failed replication, citations, metascience, scientific impact

## Abstract

With a sample of 228 psychology papers that failed to replicate, we tested whether the trajectory of citation patterns changes following the publication of a failure to replicate. Across models, we found consistent evidence that failing to replicate predicted lower future citations and that the size of this reduction increased over time. In a 14-y postpublication period, we estimated that the publication of a failed replication was associated with an average citation decline of 14% for original papers. These findings suggest that the publication of failed replications may contribute to a self-correcting science by decreasing scholars’ reliance on unreplicable original findings.

Scholars have long been concerned about the reliability and validity of scientific findings (1). In the past decade, major efforts have been made to take such concerns seriously (2) and replicate prior work (3). Recent estimates suggest that ~64% of psychology studies replicate with effect sizes ~68% as large as original reports (4, 5). These efforts can improve the signal-to-noise ratio in the scientific record, but their success relies on scholars taking notice of failed replications and subsequently reducing their reliance on nonreplicable findings. Here, we test whether failing to replicate is associated with reduced citation rates.

To date, four investigations have addressed similar questions. Although these studies produced important advances, each was limited by small samples of failed replications, and together, they forward an ambiguous picture. Two papers investigating citations of studies replicated in the Open Science Collaboration’s Reproducibility Project: Psychology (3) found that successful replication was associated with higher citations and failed replication with lower citations, but neither effect approached statistical significance (6, 7). Another examination of citations for five papers before and after failing to replicate found evidence for small declines in favorable citations and small increases in unfavorable ones (8). A fourth analysis found that papers that failed to replicate were cited more than papers that successfully replicated both before and after the replication but that 12% of postfailure citations were negative and explicitly referenced the failures (9).

These papers used unique procedures and so are not directly comparable, but the general interpretations for the relationship between replication failures and citations appear mixed. We seek to provide a clearer picture by analyzing changes in citation patterns to original papers (OPs) after publication of a failed replication paper (FRP) and with more failed replications than earlier investigations. Additionally, we adopt a different approach. Given potentially important differences between 1) papers chosen and not chosen for replication and 2) papers that successfully replicate or fail to, it is challenging to estimate the counterfactual number of citations papers with unsuccessful replications would receive absent their failures. We handle this challenge by exclusively considering papers with failed replications and comparing their average citation trends over time before and after failures to replicate, effectively treating papers with failed replications as their own control group. We can thus estimate average expected citation trends over time for this specific group of papers before and after replication failures.

## Results

### Preprocessing.

We logged citation counts (after adding 1) to normalize residuals and restricted analyses to the first 14 y postpublication (83.3% of the data) due to a substantial drop-off in data beyond that. This resulted in a total sample size of 2,919 logged citation counts (1,804 pre-FRP; 1,115 post-FRP).

### Models.

We fitted a series of hierarchical linear models (HLMs) including random intercepts for OPs. An initial model predicted logged citation counts from OPs’ number of years since publication and its first five polynomials (model 1). To account for differences in citation trends between relatively older and more recent OPs, we modeled effects of publication year (mean-centered), plus an interaction term between publication year and years since publication (model 2). We then estimated the effect of FRPs by adding an indicator coded 0 for years prior to FRP and 1 for subsequent years (model 3). This improved model fit, Δχ^2^(1) = 26.16, *P* < 0.001, ΔR^2^ = 0.01, with a significant negative slope on FRP, b = −0.17, *SE* = 0.03, *t*(2,740) = −5.12, *P* < 0.001. We then tested whether this effect increased over time by adding years since FRP (YS_FRP; scored 0 for all years preceding and the year of FRP, increasing by 1 each subsequent year; model 4). Doing so again improved model fit, Δχ^2^(1) = 119.17, *P* < 0.001, ΔR^2^ = 0.03, with a significant negative slope on both FRP, b = −0.15, *SE* = 0.03, t(2,731) = −4.72, *P* < 0.001 and YS_FRP, b = −0.09, *SE* = 0.009, t(2,742) = −10.76, *P* < 0.001.

**Fig. 1.**
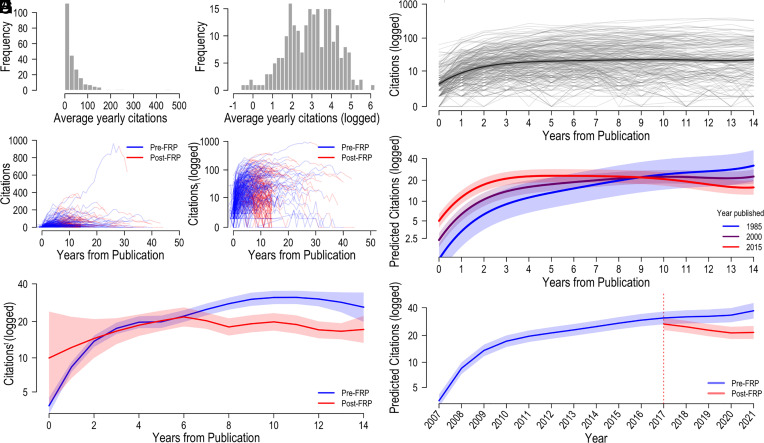
Data visualization and model output. Note: Panels *A* and *B* display distributions of raw and logged average citation counts. Panels *C* and *D* display raw and logged citation counts over time. Panel *E* displays locally estimated scatterplot smoothing (LOESS) curves for pre- and post-FRP observations. Panels *F*–*H* display model predictions from models 1, 2, and 4, respectively. Shaded regions indicate 95% CIs.

To interpret these effects, we compared each paper's predicted citations following replication failures with counterfactual predicted citations assuming that they had not failed to replicate. Reconverting predictions from logged to real units, average predicted citations were reduced from 24.09 to 20.82 following FRPs, a 14% reduction. Panel *H* of Fig. 1 plots predicted citations with and without an FRP for an OP published in 2007 with an FRP in 2017 (roughly the average paper in our data). A boot-strapped power sensitivity analysis suggested that 80% power to observe significant effects of FRP and years since FRP was achieved at sample sizes of 100 and 30 papers, respectively.

To test the robustness of these results, we explored various further model specifications using additional control variables, data, and modeling techniques (see Table 1, models 6 to 10). In one case, these changes led to the main effect of FRP becoming nonsignificant, but in all models, years since FRP remained a negative and significant predictor, suggesting a robust effect of citation trends becoming increasingly negative following FRPs.

**Table 1.** Model results

	Model 1	Model 2	Model 3	Model 4	Model 5	Model 6	Model 7	Model 8	Model 9	Model 10
Fixed effects	$\hat{\beta }$	$\hat{\beta }$	$\hat{\beta }$	$\hat{\beta }$	$\hat{\beta }$	$\hat{\beta }$	$\hat{\beta }$	$\hat{\beta }$	$\hat{\beta }$	$\hat{\beta }$
(Intercept)	1.515***	1.467***	1.466***	1.464***	1.462***	1.458***	1.513***	−1.382***	1.486***	1.841***
YSP	0.972***	0.991***	1.006***	1.019***	1.022***	1.018***	0.744***	0.971***	1.03***	1.065***
YSP^2^	−0.251***	−0.257***	−0.266***	−0.273***	−0.275***	−0.272***	−0.13***	−0.252***	−0.287***	−0.267***
YSP^3^	0.033***	0.034***	0.036***	0.038***	0.038***	0.038***	0.011***	0.033***	0.042***	0.035***
YSP^4^	−0.002***	−0.002***	0.002***	−0.002***	−0.003***	−0.002***	−0.0004***	−0.002***	−0.003***	−0.002***
YSP^5^	0.0001***	0.0001***	0.0001***	0.0001***	0.0001***	0.0001***	0.00001***	0.0001***	0.0001***	0.0001***
Published		0.04***	0.04***	0.034**	0.032**	0.032***	0.03**	0.032***	0.035***	0.062***
YSP × Published		−0.004***	0.004***	−0.001**	−0.001	−0.001**	−0.0003	−0.004***	−0.0005	−0.003***
**FRP**			−0.167***	−0.146***	−0.137***	−0.148***	−0.137***	**0.01**	−0.27**	−0.057**
**YS_FRP**				−0.094***	−0.122***	−0.095***	−0.091***	−0.045***	−0.113**	−0.066***
Replicated					0.011					
YSP × Replicated					−0.002					
Published × Replicated					0.002					
YS_FRP × Replicated					−0.008*					
Random effects	*SD*	*SD*	*SD*	*SD*	*SD*	*SD*	*SD*	*SD*		
Paper	1.055	1.056	1.05	1.042	1.044	1.039	1.066	0		
Residual	0.462	0.446	0.444	0.434	0.434	0.434	0.451	0.427		

Notes: *** = *P* < 0.001, ** = *P* < 0.01, and * = *P* < 0.05. YSP = years since publication, Published = year of publication (mean-centered), FRP = failed replication (0 = no, 1 = yes), YS_FRP = years since failed replication, Replicated = year of replication (mean-centered). Models 1 to 5 are HLMs predicting logged citation rates within the first 14 y postpublication excluding one outlying paper. Model 6 adds the outlying paper back to the dataset. Model 7 extends the time window of data used in the model to include the first 25 y postpublication (92.3% of all data). Model 8 predicts logged citations mean-centered within papers. Model 9 is a linear model using heteroskedasticity- and cluster-robust SEs. Model 10 is a generalized estimating equation model predicting raw citation counts using a Poisson error distribution and an autoregressive correlation structure (AR-M) correlation structure. Bold indicates the variables of primary interest.

## Discussion

Failing to replicate predicts declines in future citations, with this relationship increasing over time. These results suggest the possibility that scholars take notice of effects that fail to replicate and reduce their reliance on them in their own theorizing (although some might find the magnitude and speed of these corrections inadequate).

One important limitation of the present work is its observational design. OPs are not randomly assigned to be replicated, so we cannot rule out the possibility that confounding factors besides FRPs (e.g., conceptual replications, related empirical work, methodological developments, and criticisms of past scientific norms) may have produced the observed citation declines.

Another limitation is that we binarily classified failed replications based on FRP authors’ and our own subjective assessments. Results of replication attempts, however, exist on a continuum, ranging from ambiguous to strong evidence for or against an original finding. Often, it is not obvious whether a replication effort did or should cast doubt on original findings (10). Additionally, because of differences in how scholars describe replication attempts and their relative success, our Google Scholar search likely missed relevant replication efforts. Future research should test whether citation declines are steeper for papers that catastrophically failed to replicate compared to papers with only moderate or ambiguous evidence of failed replication.

We also did not assess whether OP citations were positive/affirmative vs. negative/critical. Given that we see an overall decline in citations, and some postfailure citations are negative/critical [estimates range from 3% (7) to 12% (9)], the true decline in positive/affirmative citations is likely larger than the decline we observed. Future research should explore declines in affirmative citations and how frequently FRPs are cocited with OPs. Soon, these tasks may be automatable with Artificial Intelligence.

Future work could also test whether failed replications are associated with steeper or shallower citation declines when published as part of a large-scale replication vs. a standalone replication. Large-scale replication efforts seem to attract attention, but they also tend to de-emphasize the specific papers that failed to replicate. Thus, large-scale projects may have a relatively larger or smaller impact on the perceived reliability of OPs.

Although matching papers with and without failed replications presents some challenges, future work should seek to replicate our results using alternative approaches that compare citation patterns among papers that failed to replicate to two additional classes of papers (ideally, relatively matched in other qualities, such as initial impact): 1) papers that successfully replicated and 2) papers with no known replication attempts. Our results suggest that papers that fail to replicate likely receive fewer citations post-failure-to-replicate relative to these classes of papers.

Recent replication efforts may indeed be contributing to a more self-correcting science.

## Materials and Methods

### Open Science Statement.

This study was not preregistered. Data and analysis code are available here: https://osf.io/8ustx/ and https://osf.io/8ustx/?view_only=a6e84a79e33f4a848d5ebb97d4936fc2. See *SI Appendix* for our expanded Open Science Statement describing analytic decisions as well as Procedure details sufficient for replication.

### Procedure.

In late 2021, we collected FRPs from major replication projects (n = 154) (5) (11), and a Google Scholar search for “direct replication,” “failed,” and “psychology,” restricting the range from 2012 (the start of the replication crisis) to 2021 (n = 120). We collected all associated OPs and excluded duplicates and one outlying OP published 32 y earlier than any other. Our final sample included 228 unique OPs published between 1978 and 2021 that later failed to replicate. In early 2023, we used Google Scholar to code the number of citations each OP received every year after publication, ensuring that data were complete until the end of 2022, so each OP had at least 1 y of postfailure citation data.

## Supplementary Material

Appendix 01 (PDF)

## Data Availability

csv data have been deposited in OSF (https://osf.io/8ustx/?view_only=a6e84a79e33f4a848d5ebb97d4936fc2) (12). All other data are included in the manuscript and/or *SI Appendix*.
