# Supplementary material for: Failing to replicate predicts citation declines in psychology
Source: Proc Natl Acad Sci U S A. 2023 Jul 10;120(29):e2304862120. doi: 10.1073/pnas.2304862120 (PMC10629524; doi:10.1073/pnas.2304862120)
Supplement: Supplementary file 1 — Appendix 01 (PDF) [file pnas.2304862120.sapp.pdf]

Supplemental Methods for Failing to Replicate Predicts Citation Declines in Psychology

Clark, Connor, & Isch, 2023

Open Science Statement (Expanded)

Readers should note that in the present project, analytic decisions were made that were both data-dependent and results-dependent. For example, the decision to restrict analyses to the first 14 years post publication was made only after looking at the data and realizing that this was the window in which we had a good-sized sample of pre- and post-FRP observations, and the decision to log citation counts was made after seeing the strong positive skew of the raw citation counts. The decision to model the average time trend of citations via the first five polynomials of years since publication was made due to the inclusion of the sixth-order polynomial failing to significantly improve model fit. And the decision to model differences in average citation trends between older and more recent papers via year published (mean-centered) and its interaction with years since publication was made after the inclusion of an interaction between year published (mean-centered) and years since publication squared failed to significantly improve model fit.

However, we did not make any analytic decisions based on their influence on our conclusions. Indeed, we believed that the results would be of interest to the scientific community regardless of what we found. We have included all data and variables collected in the present project (except the exclusions reported in the text), and have tried to provide a comprehensive account of our results via the various robustness checks reported and described in our manuscript. We also provide both our data and code for anyone who would like to fit alternate models to the data.

Procedure (Expanded)

In Fall of 2021, we collected papers that reported failed replications (FRPs) from the State of Replicability Subproject (1) and the Replicability, Robustness, and Reproducibility in Psychological Science Project (2), including only papers that were coded as failing to replicate the original work (n=154). Next, we conducted a Google Scholar search for "direct replication", "failed", and "psychology", restricting the range from 2012 (the start of the replication crisis) to 2021. As of September 8, 2021, this search returned 2,870 hits. We scanned each paper to determine whether it indeed reported a failed direct replication in psychology with a stopping rule of 100 consecutive false positives. This search returned an additional 120 FRPs. See the final paragraph of this expanded procedure section for discussion of the challenges of classifying papers as FRPs.

We then collected all original papers (OPs) associated with each FRP and excluded all duplicates and cases where multiple teams assessed the same OP. We also excluded one outlying OP (3) published 32 years earlier than the next oldest OP. Our final sample included 227 unique OPs published between 1978 and 2021 that later failed to replicate.

Using Google Scholar, we coded the number of citations each OP received every year after publication. This process was accomplished as follows. First, we searched for each paper in Google Scholar. After identifying the correct publication, we determined if any of the authors had created a Google Scholar account. If they had, we navigated to that scholar's profile and again identified the article of interest. The article's page within the author's profile contains a bar plot with the number of citations that the paper received every year since publication; we scraped these values to determine the number of citations that the article had received each year. In instances where no author had an associated google account, we navigated to the "cited by" page

associated with the original paper. We limited the search window to one-year intervals, starting with the year that the article was published and ending in 2022. This returns every item published in a given year that references the original paper. We reported the total number of items found for each one year interval. With these methods, we identified the number of items that cite each OP within the Google Scholar database.

Note that, in many cases, it was obvious whether a direct replication should be considered a failed replication because the replication effects were near zero or even in the opposite direction. However, it was not always obvious. For example, sometimes replication papers replicated some effects and not others, or found a statistically significant effect that was substantially smaller than the original, or found a non-significant effect that was only somewhat smaller than the original, or replicated the effect in some studies but not others. We resolved ambiguous cases in one of two ways: either we emailed the lead author of the replication paper and asked whether they would consider their results a failed replication, or two members of the research team (who were not involved in data analysis) agreed on whether the results should be considered a failed replication. But these determinations are at least somewhat subjective, and different scholars likely hold different standards for what should be considered a failed vs. successful replication (see, e.g., 4). Indeed, the evidence for or against original findings exists on a continuum, and different scholars likely update their confidence to different degrees at different places along that continuum. It thus seems likely that some scholars may disagree on whether certain papers should or should not have been included in our dataset and whether certain replication efforts should or should not cause scholars to update their beliefs about OPs.

Statistical Modeling (Expanded)

Overall Time Trend Model. To account for the nested structure of our data, we fitted hierarchical linear models (HLMs) with the lme4 and lmerTest packages in R and included random intercepts for OPs. Our first step was to fit a model for the overall average time trend for citation rates in the first 14 years following publication. In the absence of any strong theoretical priors about the nature of this trend, we used a data-driven approach. First, we first predicted logged citation counts from a linear trend of years since publication (YSP; i.e., for a paper published in 2008 YSP is scored 0, 1, and 2, for the years 2008, 2009, and 2010, respectively). Then, we iteratively added higher order polynomials of years since publication (YSP^2, YSP^3, YSP^4, etc) until their inclusion no longer improved model fit (assessed via analysis of deviance/Chi squared tests). This process resulted in an initial model including all polynomials up to YSP^5 (Model 1 in Table 1, panel F in Figure 1), which fit the data significantly better than a model including all polynomials up to YSP^4, $\Delta\chi^2(1) = 21.18$, $p < .001$. Adding a sixth order polynomial YSP^6 did not significantly improve fit, $\Delta\chi^2(1) = 1.68$, $p = 0.19$.

Controlling for Publication Date. When comparing pre- and post-FRP citation trends within OPs' first 14 years post-publication, an important potentially confounding factor is that within that window, older papers account for relatively more/less pre-FRP/post-FRP observations, compared with more recent papers. As a result, it is possible to observe a difference in citation time trends between pre- and post-FRP citation trends that is not due to an effect of FRPs, but is instead due to differences in citation trends between relatively older and more recent papers. To guard against this, we modeled changes in average citation trends across time by modeling effects of each OP's year of publication (mean-centered), plus an interaction term between year of publication and years since publication (Model 2 in Table 1). These additions significantly improved model fit, $\Delta\chi^2(2) = 191.72$, $p < .001$. Panel G of Figure 1 in our

manuscript visualizes predicted citation trends for papers published in 1985, 2000, and 2015, with the interaction pattern suggesting that older OPs in our dataset tended to be cited less often immediately after publication than more recent papers, but continued to increase in citations for longer than more recent papers. Adding an interaction term between year published and YSP^2 did not significantly improve model fit, $\Delta\chi^2(1) = 0.08$, $p = 0.77$.

Modeling Effects of FRP. We next estimated a simple main effect of FRPs by adding as a predictor an indicator coded 0 for years prior to FRP, and coded 1 for the year of FRP and subsequent years (Model 3 in Table 1 in our manuscript). We then tested whether the effects of FRPs might accrue over time by adding as a predictor years since FRP (YS_FRP; scored 0 for all years preceding FRP and the year of FRP, increasing by 1 for each subsequent year; Model 4 in Table 1 in our manuscript). Adding the squared term of YS_FRP (YS_FRP^2) did not improve model fit, $\Delta\chi^2(1) = 2.73$, $p = 0.10$.

Power Sensitivity Analysis. To assess the sample size necessary for 80% power to observe (1) the main effect of FRPs and (2) the increased effect of FRPs over time, we conducted a bootstrapped power sensitivity analysis. First, we selected a range of sample sizes to test (from 10 to 230 increasing in increments of 10). Then, at each sample size, across 1000 iterations we randomly selected with replacement papers from our dataset, and for each new dataset re-fitted Model 4, and recorded slopes and p values for the predictors FRP and years since FRP. For each effect at each sample size, estimated power was the proportion of iterations in which slopes were both negative and statistically significant at an alpha level of 0.05. For the main effect of FRP 81% of slopes were negative and significant at a sample size of 90 papers. For the effect of years since FRP, 85% of slopes were negative and significant at a sample size of 40 papers.

Robustness Checks. To test the robustness of these results regarding the FRP and YS_FRP, we explored various further model specifications using additional control variables, data, and modeling techniques. Model 5 included as a predictor OPs' year of replication (mean-centered), as well as three interaction terms: an interaction between year of replication and years since publication (YSP), an interaction between year of replication and years since failed replication (YS_FRP), and an interaction between year of replication and year published. Model 6 re-fit Model 4 but included the outlying OP (Asch, 1946). Model 7 again re-fit Model 4, but excluded the time outlying OP and extended the time window to the first 25 years after publication (this meant including 92.3% of all observations excluding the time outlier). Model 8 re-fit Model 4 but predicted logged citation counts mean-centered within OPs, thus removing any overall between-paper differences in average citations. Model 9 re-fit model 4 but instead of fitting an HLM, fitted a linear model with heteroskedasticity- and cluster-robust standard errors clustered within OPs. Model 10 re-fit model 4 but instead of predicting logged citation counts, used a Generalized Estimating Equation (GEE) model predicting raw citation counts using a Poisson error distribution and an AR-M correlation structure.

References

1. K. S. Corker, Allard, A., Nosek, B. A., & Moshontz, H., State of replicability. https://osf.io/547ws/.
2. B. A. Nosek *et al.*, Replicability, robustness, and reproducibility in psychological science. *Annual Review of Psychology* **73**, 719-748 (2022).
3. S. E. Asch, Forming impressions of personality. *The Journal of Abnormal and Social Psychology* **41**, 258-290 (1946).
4. A. Etz, Vandekerckhove, J. A Bayesian perspective on the reproducibility project: Psychology. *PloS One* **11**, e0149794 (2016).
